# International medical students’ adaptation to university life in Turkey

**DOI:** 10.5116/ijme.5e47.d7de

**Published:** 2020-02-28

**Authors:** Nilufer Demiral Yilmaz, Hatice Sahin, Aylin Nazli

**Affiliations:** 1 Ege University School of Medicine Department of Medical Education, Izmir, Turkey; 2 Ege University School of Letters Department of Sociology, Izmir, Turkey

**Keywords:** International medical student, adaptation, coping strategy, mixed-method

## Abstract

**Objectives:**

The aim of this study is to determine the adaptation process of international medical school students to university life in Turkey.

**Methods:**

The mixed method design study, including all the international students (n=127) studying at Ege University School of Medicine, is employed. Qualitative data were collected from 23 students selected by purposive sampling technique. Two instruments were utilized for collecting data: Student Adaptation to College Questionnaire (SACQ) and Brief COPE. Focus group interviews were performed for qualitative data collection.

**Results:**

The mean SACQ score of the medical students was found to be 407.44 (SD=68.29). The Academic Adjustment category included the themes of educational goals, different studying habits, accommodation, academic advisor, and scholarship. The Social Adjustment category included the themes of social interaction and support, differences, longing for family, discrimination and its effects on social life. The Personal/Emotional adjustment category included themes of psychological and physiological health problems. The Goal Commitment/Institutional Attachment category included themes of academic and social adaptation as well as the student’ communication with education management.

**Conclusions:**

This study identifies to light critical issues in supporting international students with adaptation problems to university life in Turkey. It is clear that revising the content of education programs to enable international exchange is not sufficient enough by itself to meet the needs of international students.

## Introduction

University life is a challenging period for students since it requires effort in different areas. These efforts include integration to academic and social life at a university, dealing with newfound freedoms, becoming more individualized, and establishing social relations with male and female peers, Higher education is also a time period where cultural adaptation is experienced.^1^^-^^8^ There are various theories and views used in defining adaptation to a new cultural environment, a few theories commonly explain cultural adaptation to this new environment. One of them is Social Isolation Theory which not only describes an individual’s physical deportation from the homeland but also from a series of rights, rules, and social interaction patterns that the individual is accustomed to. Another one is Cultural Shock Theory which analyzes the effects of living in an international environment on an individual’s overall health. The last one would be Cultural Change Theory defining the idea that the stressful life brought about by cultural change influences also the personalities of other family members. One or more of these theories may apply to any case involving cultural adaptation.

Lysgaard’s U curve, Oberg’s seven-step acclimation process, Gullahorn’s W curve, and Peter Adler’s five-step developmental process are among the approaches used to explain cultural adaptation. Lysgaard has stated that the adaptation processes of individuals who have to live in a different culture change in time. This approach, which was the first used to explain cultural adaptation, is called the “U Curve”. The “U Curve” approach has four stages of adaptation; namely the honeymoon, culture shock, adaptation, and double culture.^9^ When an international student first goes to a country away from his/her family and customs, everything about the new culture feels exciting and interesting. Getting to know the new environment is at first fun for the student.  This phase lasts approximately for two months. As time passes, the academic and social requirements of higher education cause the student to have a difficult time in fulfilling these requirements; therefore, adapting fully-or as much as needed- resulting in a culture shock that lasts for about six months. By the end of the cultural shock phase, the student understands the characteristics of the culture he/she is in and starts to feel that this new environment is home. In the “U Curve” process, which lasts for approximately 40 months, the student adapts to the new culture.^10^ After a little more time, the student can effectively execute actions and relations in the new environment, and move freely between the new culture and the student’s own culture. Thus, within approximately 48 months of time, the process is completed as the dual culture phase.

The phases of cultural adaptation form a special period that requires time and adequate coping strategies to deal with emerging stress. According to previous researches, some students respond positively to such transition problems and increase their level of adaptation. In the cultural adaptation process, the student uses various coping strategies to deal with the stress and problems that may emerge. However, if the student’s coping strategies are not sufficient, adaptation to the new culture would be more difficult.^11^^-^^18^

The disease-health patterns of different countries affect the content of medical education programs, their languages, and the type of communication between doctor and patient. The undergraduate medical education programs have a different character compared to other college programs in this regard. These differences make cultural adaptation harder especially for international students.^1^^-^^4^^,^^7^ For this reason, the adaptation of international medical students to university life in Turkey should be taken into consideration, and an appropriate student counseling system should be established for these students. The aim of this study is to determine international medical school students’ adaptation to university life in Turkey.

## Methods

### Study design and participants

This study adopts a mixed-method approach which combines elements of qualitative and quantitative research approaches.^19^^-^^24^ The Explanatory Design (quantitative>qualitative) as defined by Creswell is used in the study, and qualitative data is collected after quantitative data is collected and analyzed.^19^

The study consisted of all of the international students (n=127) studying at Ege University School of Medicine. Among the students, 83.46 % (n=106) participated in the study, while 21 students did not agree to participate.

The mean age of the students was 23.33±2.86 (min=17–max=35). 68% (n=73) of the students were male, 99% (n=105) were single, 63.20% (n=67) lived in dormitories, 72.60% were accepted to the university via exams, and 51.90% learned Turkish in Turkey. The average duration of study in medical school was 4.35±2.03 years (Table 1).

**Table 1:** The participation of the students in the study

Class	Students	Students reached	Reach (%)
1	34	16	66.67
2	30	24	100
3	20	19	95
4	29	24	85.71
5	17	15	88.24
6	14	8	57.14
Total	144	106	83.46

**Figure 1:**
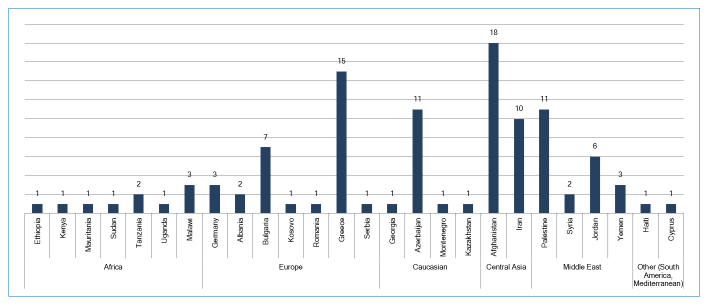
The distribution of students according to the region and countries they come from

Students came from seven different regions in the world to study at Ege University School of Medicine.  28% came from Europe, 26% from Central Asia, 20% from the Middle East, 13% from the Caucasus, 9% from Africa, and 1% from either South America or the Mediterranean (Figure 1).

The sample of the study was determined according to the most widely used method called ordered quantitative-qualitative technique suggested by Kemper and colleagues.^25^ With this sampling technique, the last sample used in quantitative phase determines the sample selection in the qualitative phase. This study aimed at collecting quantitative data from all 127 international undergraduate students at Ege University School of Medicine. In order to obtain qualitative data, 23 students were selected from the students in the quantitative sample^26^ (country, class, gender) by the purposive sampling technique; and then they were invited to attend focus group interviews.

Ethical approval from the Ethics Board of Ege University School of Medicine was obtained prior to this research. The students were also informed on the objective and methods of the study, and they were requested to sign a written consent.

### Data collection

A mixed-method data collection approach using both interview and questionnaire research techniques is adopted to achieve the research aim.^19^^-^^20^ For this purpose, quantitative and qualitative data are collected, analyzed, and presented in the study. The quantitative data of the study was collected using the Student Adaptation to College Questionnaire and the Brief COPE. Qualitative data was collected using focus group interviews.

The Student Adaptation to College Questionnaire (SACQ), developed by Baker and Siryk, was found to be reliable and valid.^27^ The questionnaire consists of 67 items scored on a 9-point Likert type scale (1: doesn’t fit me at all, 9: fits me perfectly). The total score changes between 67 and 603. A higher total score shows better adaptation to university life. The characteristics of the four subscales, the academic adjustment, the social adjustment, the personal/emotional adjustment, and the goal commitment/institutional attachment, are as follows: first, the Academic Adjustment subscale consists of 24 items regarding academic motivation, academic effort, academic performance, and being pleased with the academic environment.

The score that can be taken from this subscale changes between 24 and 216. Second, the Social Adjustment subscale consists of 20 items. The items are about success in social activities, the social aspects of the university environment, relations to roommates and opposite sex, and being homesick. The score that can be taken from this subscale changes between 20 and 180. Third, the Personal/Emotional Adjustment subscale consists of 15 items regarding psychological and physical health. Scores vary between 15 and 135. And fourth, the Goal Commitment/Institutional Attachment subscale consists of 15 items. These evaluate emotions and levels of satisfaction regarding university as an institution. Scores vary between 15 and 135.^28^

The Brief COPE is the short form of the COPE scale developed by Carver, Scheier, and Weintraub.^29^ The scale consists of 18 items scored on a 4-point Likert type scale (1: I never do that, 4: I do that excessively). The Brief COPE consists of 14 subscales, namely   Active Coping, Planning, Use Of Instrumental Support, Use Of Emotional Support, Venting, Self-Distraction, Positive Reframing, Denial, Acceptance, Religion, Substance Use, Behavioral Disengagement, Humor, and Self-Blame.^30^ Scoring gives information on which of the 14 different coping strategies is used more frequently. The scale was adapted to Turkish and tested for validity and reliability by Tuna.^31^

Moreover, focus group interviews were used for collecting qualitative data. A total of two focus group interviews were conducted, and data collection ceased when saturation was reached. Each focus group interview was carried out with 13to 15 students from diverse genders, nationalities, and years of study. Each interview took approximately 75 minutes. During the interviews, students were asked questions about their experiences regarding academic, social, emotional, and institutional adaptation. All interviews were audio recorded.

### Procedure

The students filled out the Student Adaptation to College Questionnaire and the Brief COPE in a single session in March 2015. Self-administered questionnaire technique was used in this study. Focus group interviews’ were conducted in April 2015. Focus groups were audio-recorded, transcribed verbatim, and analysed for content.

### Data analysis

Descriptive statistics were used to report the findings. In order to make comments on the adaptation process to university, the Maximum Score Approach Rate, the ratio of approximity to the maximum point that can be obtained from the Student Adaptation to College Questionnaire and its subscales, is calculated. In Maximum Score Approach Rate calculation, the formula [(Observed score/expected maximum score) x 100] was used. A higher Maximum Score Approach Rate showed better adaptation.^32^ Quantitative data were analyzed using the SPSS for Windows (SPSS, Inc. IBM) version 18.0.

The discussion from focus groups was audio-recorded and transcribed verbatim. Transcripts were independently coded by two of the authors at the early stage of the coding process. Afterwards, the authors compared data and reached an agreement between the identified codes and refined the coding frame. The themes and sub-themes were identified and findings were reported accordingly with the aid of illustrative quotes. The themes were named according to the subscales of The Student Adaptation to College Questionnaire. Qualitative data analysis was performed using the NVivo 10 program. The findings of quantitative and qualitative data were embedded in order to be interpreted. While embedding, qualitative data codes were categorized according to the subscale titles of the Student Adaptation to College Questionnaire and the Brief COPE. Each code was related to one or more categories. The categories were then grouped under certain themes. Thus, the themes stressed by both quantitative and qualitative data have made it possible to interpret data regarding adaptation to university and coping strategies together.

## Results

The mean Student Adaptation to College Questionnaire score of the students was found to be 407.44 (SD=68.29). The mean subscale score were as follows: Academic Adjustment: 130.59 (SD=26.20); Social Adjustment: 102.14 (SD=20.03); Personal/Emotional Adjustment: 77.31 (SD=21.23); Goal Commitment/Institutional Attachment: 89.29 (SD=17.98). In this study, the items in the social adjustment subscale had the lowest mean score, while the items in the Goal Commitment/Institutional Attachment subscale had the highest mean score.

The Maximum Score Approach Rate of the entire scale was 67.57%. The Maximum Score Approach Rate of the subscales were as follows:  Academic Adjustment 60.46%; Social Adjustment 56.75%; Personal/Emotional Adjustment 57.27%; and Goal Commitment/Institutional Attachment 66.14% (Table 2).

Adaptation of the students to the university life was found to be on a satisfactory level (Maximum Score Approach Rate=67.57%).

**Table 2:** The Student adaptation to college questionnaire and subscale mean scores and maximum score approach rate

Subscales	Item number	Expected score	Observed score				
		Min-max	Mean (SD)	Min.	Max.	Maximum Score Approach Rate (%)	
Academic adjustment (AA)	24	24-216	130.59 (26.24)	63.00	189.00	60.46	
AA motivation	6	6-54	38.73 (7.24)	17.00	54.00	71.72	
AA application	4	4-36	22.18 (6.26)	7.00	34.00	61.61	
AA performance	9	9-81	42.27 (11.34)	20.00	69.00	52.19	
AA academic environment	5	5-45	26.42 (5.37)	8.00	38.00	58.70	
Social adjustment (SA)	20	20-180	102.14 (20.03)	48.00	153.00	56.75	
SA general	7	7-63	37.63 (8.40)	19.00	58.00	59.73	
SA other people	7	7-63	36.06 (8.33)	10.00	55.00	57.23	
SA nostalgia	3	3-27	15.67 (5.52)	3.00	27.00	58.04	
SA social environment	3	3-27	12.78 (4.63)	3.00	27.00	47.34	
Personal / emotional adjustment (PEA)	15	15-135	77.31 (21.23)	30.00	125.00	57.27	
PEA psychological	9	9-81	45.28 (13.95)	12.00	75.00	55.90	
PEA physiological	6	6-54	32.03 (8.83)	10.00	52.00	59.31	
Goal commitment / institutional attachment (AT)	15	15-135	89.29 (17.98)	53.00	130.00	66.14	
AT general	3	3-27	21.23 (5.47)	7.00	27.00	78.62	
AT this college	4	4-36	25.99 (7.58)	7.00	36.00	72.20	
The Student Adaptation to College Questionnaire	67	67-603	407.44 (68.29)	273.00	581.00	67.57	

When adaptation to university life was evaluated on a subscale level, they were found to be listed from positive to negative as Goal Commitment/Institutional Attachment, Academic Adjustment, Personal/Emotional Adjustment and Social Adjustment. This may be interpreted as students’ prioritizing their training goals and disregarding social adaptation.

On the other hand, the Brief COPE subscale mean scores were as follows; Planning 6.51 (SD=1.28); Acceptance 5.85 (SD=1.28); Active Coping 5.84 (SD=1.35); Positive Reframing 5.67 (SD=1.28); Self-Blame 5.58 (SD=1.51); Self-Distraction 5.58 (SD=1.51); Use of Instrumental Support 5.38 (SD=1.58); Venting 5.11 (SD=1.46); Religion 5.07 (SD=1.84); Use of Emotional Support 4.95 (SD=1.49); Humor 4.69 (SD=1.66); Denial 3.92 (SD=1.52); Behavioral Disengagement 3.87 (SD=1.56); Substance Use 3.06 (SD=1.73) (Table 3).

The highest mean score in the Brief COPE belonged to the Planning subscale 6.51 (SD=1.28), and the lowest belonged to the Substance Use subscale 3.06 (SD=1.73).

### Focus group interviews

The students stated that they chose medical school since “being a doctor was an important occupation for human life.” They were admitted to Ege University through the exams they took in their own countries or the International Student Entrance Exam in Turkey.

One hundred seventeen codes were extracted in the analysis of qualitative data. When these 117 codes were categorized (15) according to the Student Adaptation to College Questionnaire subscales, 50 were related to Academic Adjustment, 53 to Social Adjustment, 34 to Personal/Emotional Adjustment, and 25 to Goal Commitment/Institutional Attachment. A single code could be included in multiple categories. When the codes were categorized according to the subscales of the Brief COPE, the subscales were related to the following number of codes: Active Coping 11, Planning 23, Use of Emotional Support 17, Acceptance 9, Behavioral Disengagement 2, Substance Use 1, Venting 3, and Positive Reframing 3. The themes related to the categories were determined.

**Table 3:** Brief COPE subscales and score averages

Subscales	Item number	Min-max score	Mean (SD)
Planning	14-25	2-8	6.51 (1.28)
Acceptance	20-24	2-8	5.85 (1.28)
Active coping	2-7	2-8	5.84 (1.35)
Positive reframing	12-17	2-8	5.67 (1.28)
Self-blame	13-26	2-8	5.58 (1.51)
Self-distraction	1-19	2-8	5.58 (1.51)
Use of instrumental support	10-23	2-8	5.38 (1.58)
Venting	9-21	2-8	5.11 (1.46)
Religion	22-27	2-8	5.07 (1.84)
Use of emotional support	5-15	2-8	4.95 (1.49)
Humor	18-28	2-8	4.69 (1.66)
Denial	3-8	2-8	3.92 (1.52)
Behavioral disengagement	6-16	2-8	3.87 (1.56)
Substance use	4-11	2-8	3.06 (1.73)

The Academic Adjustment Category included the themes of having training goals and being resolute in studying, having different studying habits, accommodation and transportation conditions, not being able to see an academic advisor at school, and scholarship. The Social Adjustment Category included the themes of social interactions and support, differences, missing one’s family, discrimination, and their effects on social life. In addition, The Personal/Emotional Adjustment Category included the themes of psychological and physiological health problems as well as health insurance. And finally, the Goal Commitment/Institutional Attachment Category included the themes of academic and social adaptation as well as the students’ communication with education management.

The qualitative data findings, which were embedded into quantitative data, are summarized below by using the determined themes.

### Academic adjustment

Students stated that there were positive and negative factors affecting academic adaptation. The factors that positively affected the process can be listed as having education goals for the purpose of becoming a good physician and being resolute in studying.

“… of course, we go through hardships, but I think that anyone who leaves his/her country and comes here has a goal and tries to achieve it every day…” (Student 1, Male)

When the factors affecting academic adjustment negatively were examined, the prioritized ones were seen to be the Turkish education system being different from their studying habits, of accommodation and transportation, not being able to reach their counselors at school, getting a scholarship with a stipulation of success, and fear of losing the scholarship.

The international students face a different education system from that of the country they come from. In the medical school education program for them courses are hard, exams are frequent and different in method, and student success is tied to attendance. These students, who used to be successful in their countries, have to study much harder than their peers

to academically adapt to their new school. When the language problem is added to this, adaptation becomes even more difficult. Students stated that they have gotten used to heavy courses and the education system over the years.

“… sometimes you go to classes and they are hard so you have to study like we have this system back there where we have to make a transition from” (Student 1, Male)

“I think the curriculum is too heavy… We see theoretical courses from 8.30 AM to 5.00 PM… I don’t repeat the daily courses only once, but have to 2 or 3 times to remember so much…” (Student 5, Female)

“… I didn’t understand the word but it was Arabic, which is my mother tongue, still it could mean something else…” (Student 4, Male)

Most of the students choose to live in an apartment. Apart from creating a free environment, having responsibilities regarding housework affects academic adaptation negatively. Students who stay at dormitories report different problems concerning living conditions. For instance, not having a suitable living and studying environment in crowded dorm rooms is the most important factor in challenging academic adaptation.

“Since a house gives more freedom” (Student 4, Male)

“I stayed at a dorm for 3 years. We were 6 people in the room, and when it is midnight you want to sleep since you have to wake up the next morning; but people don’t want to sleep, they want to read and they make noises. When I wake up in the morning, I open my wardrobe slowly so that I don’t make a noise since others sleep. We were 6 roommates.” (Student 5, Female)

Some students stated that since time lost in transportation shortens studying time, living near the school was vital with regard to academic adjustment.

“I prefer the dorm since we need time and living in an apartment is a loss of time since you cook and do everything. Everything is ready for you in the dorm.” (Student 16, Female)

“… classes begin at 8.30 AM and they continue until 5.00 PM, and I stay at a dorm, and have to come here every morning from ... We encounter a lot of hardships…” (Student 1, Male)

“Other than apartment or dorm, it is important for us to be close to school.” (Student 4, Male)

International students also want to meet their academic counselors to solve the problems they face regarding academic adjustment. The students stated that whenever they encounter a problem about courses, they try to reach their counselors but these efforts came fruitless, and that compared to other universities, they couldn’t benefit from their counselors.

 “… I have never seen him (my counselor). We don’t know who to go to when we have a problem. I need to reach him, and tell him my problem somehow.” (Student 13, Male)

“… I couldn’t take my exam this semester… I tried to reach my counselor twice. One time, he went to Istanbul, and I tried to get his phone number… I tried to reach him twice.” (Student 11, Male)

“… you e-mail him (the counselor), but there is no answer, you can’t get past his secretary.” (Student 7, Male)

Moreover, the stipulation of success attached to scholarships creates pressure for students. In case of academic failure, the students state that their scholarships would be cancelled and they would have to return to their countries. This possibility creates certain anxiety and makes them spend most of their time studying; also damaging their social and personal/emotional adjustment.

“… we have to study, if we don’t pass, our scholarships are cut…” (Student 1, Male)

“… I want to finish school I don’t want my scholarship to be cut, so I try hard” (Student 3, Male)

“If you fail two years in a row, your scholarship is cut; so you have nothing to do, but study more and pass…” (Student 2, Male)

### Social adjustment

Social relationships and support, differences in language, culture, race/color, politics, and the education system, longing for family, and exposure to discrimination and its effect on social life are prominent factors affecting social adjustment. The students reported that they wanted to participate in social activities, but due to the fear of academic failure or losing a scholarship, they chose to study instead of participating in activities. They also stated that they received invitations to social activities from Turkish students and that they received support for compensating for lost time from Turkish students. Moreover, some international students even reported that education goals were more important than their health status and longing for family.   

“(Turkish students) sometimes say ‘Come take the study notes from me so it will take less time (to study)’. But, they also say come with us (to various activities).” (Student 4, Male)

“In my spare time, I miss (my family). But, during the examination period I do not miss them because of stress, I want to pass the exam. If you fail for two years in a row, they cut the scholarship. I need to study more and pass the exams, there is nothing to do.” (Student 16, Male)

“I think of both options if I fail; I want to finish school and I want to keep my scholarship so I put in a lot of effort” (Student 17, Male)

“I had a toothache, but I could not visit the dentist because I had to study.” (Student 8, Female)

It was observed that students received support both academically and socially from international or Turkish students, which is a positive finding pertaining to the social relations and support domain.

“I failed in my first year, than I passed the make-up exams. ….. my friend (said) we’ll study together, I received some help, (my friends) helped, I was studying with them. I was making copies of my Turkish friends’ study notes.” (Student 15, Female)

“…… an international friend of mine came and said that “I did not do it, will you help me?” So I thought if I don’t help internationals then who will help me, I should help him, so others will help me too, so I gave.” (Student 12, Male)

As to the social relations and support domain, factors that positively affect social adjustment were often related to relationships among students, while factors that negatively affect social adjustment came from lecturers.

Another factor affecting social adjustment was the differences. Students suffered from various adjustment problems associated with differences in language, culture, race/color, politics, and the education system.

“Language (our biggest personal adjustment problem).” (Student 4, Male)

“(Our biggest personal adjustment problem) Culture. Jokes. We do not understand their jokes. I want to make a really good joke but they do not understand.” (Student 8, Female)

“(Our biggest personal adjustment problem,) it is color for us, and race. Nothing would change even if you live here for 20-30 years.” (Student 7, Male)

“(Our biggest personal adjustment problem) especially being black in Turkey.” (Student 1, Male)

“Politics. We are Arabs. Something happened between Turks and Arabs in the past. You know, in the past. We should not feel like we have to (continue) this. (They say) You Arabs sold us out. Alright, they sold you out. I don’t care. Do I have to continue this?” (Student 4, Male)

“(Our biggest personal adjustment problem) Prejudice, prejudice never disappears.” (Student 20, Male)

“Every country has a system; the system here is a new system. I was intimidated (when I first came). (Simulated patient interview) took 30 minutes. I knew nothing (about the system). I forgot to stop the (camera) recording. I knew nothing; I thought that our lecturer was going to stop the camera recording. They did not show us (how to do it). Then we got used to it.” (Student 16, Male)

“I did not encounter any hardships in social adjustment. (I did not experience) language related problems. I come from the Balkans. I have relatives here. I even have more relatives than Turkish students. I am more social than most Turkish students.” (Student 18, Male)

One of the factors affecting social adjustment was also longing for family. Family is most missed during holidays. Students stated that activities of daily life (cooking, cleaning, and laundry) increased their longing for family, and that they may not even see their families for years due to financial problems.

“(My most important personal problem) missing my family. A student here would just go (when he misses his family). Bus, plane, it does not matter. Second, if he is short on money, (his family) can send him money. This is not the case for us. Because I call (my father when I need money). My father will go to the bank there, send (money). The process is long; there are problems like that.” (Student 18, Male)

In the holidays, everyone goes home, and I stay here. It has been two years (since I last visited home) (Student 15, Male).

“I miss my mother the most when I am cooking. I always had my breakfast and lunch ready when I went to school. Now I go and prepare it myself. It is very difficult.” (Student 13, Male)

“During the exams, you are stressful, but you want to study. You cannot. So you think of home more. For me, at least (this is the way it is).” (Student 5, Female)

“(Missing the family) happens most during holidays. For me that’s most important. It is not a problem for me, I just want my mother to be happy. My mother gets emotional. I can control myself. She knows I cannot go (for the holiday), and I can control myself when I talk to her, but she cannot.” (Student 12, Male)

“During holidays, everyone packs. But, I cannot go anywhere.” (Student 7, Male)

“Everyone leaves with luggage and goes to their families during make-ups. I just look!” (Student 4, Male)

It was determined that the students were exposed to prejudice and/or discriminating attitudes by lecturers in particular.

“In my first year, we were having an exam in the microscopy class. I did not understand one word on the exam. The word was Arabic. Arabic is my mother tongue, but it may have a different meaning. I called the lecturer and said “Does it mean this or does it have another meaning”. The lecturer said “Are you an international? You come here with scholarship (with money)”. (The lecturer said) why my people cannot get education, why are you here?” (Student 4, Male)

“… some lecturers are really good. They like internationals. They always understand you when you talk. They explain things to me when I don’t understand them. But, some lecturers are not that interested. There was a lecturer in my first year. I cannot remember his name. but he was male. (During the exam) I saw a question written by him. I went to him and said “I do not understand this, what does it mean?” He gave such a stern answer that I never liked him.” (Student 1, Male)

I don’t know how true it is but they don’t like strangers in our university. “You came down with a parachute, taking scholarship from us” they say with prejudice. It’s really sad. I had already gotten the points for medical school back home, and I wouldn’t have come if I hadn’t wanted to… How correct is such a welcome?!” (Student 12, Male)

“There was a blood draw skills lesson during the internal diseases internship. The patient said “ (since I was African) I don’t want her to draw blood” I got very sad and demoralized.” (Student 15, Female)

### Personal/emotional adjustment

It was found that when it came to personal/emotional adjustment, the psychological and physiological health issues encountered by the students, and health insurance coverage in taking care of these problems were important.

The international students stated that, psychologically, they experienced exam stress as well as fear of academic failure, scholarship cuts, and having to return to their country.

“I don’t get stressed during exams at all, but in the first years I used to get so stressed, I would live with the stress all year.” (Student 16, Male).

“I get affected by stress. I wasn’t overweight, but I lost 5 kilos since starting school, and I can’t gain them back.” (Student 15, Female)

“We had a friend who became schizophrenic.” (Student 13, Male)

“Two of our friends became schizophrenic, we had to send them back home. One was a senior and one was doing Masters.” (Student 12, Male)

“I had to study till morning in the first years a couple of times before I got used to exams. Since I had to study a lot, I would have fatigue.” (Student 18, Male)

The students also stated that, besides psychological problems, they have experienced physiological health problems, too; especially because of exam stress.

“I can’t eat from stress and get diarrhea during exam weeks.” (Student 15, Male)

“Me too, I get diarrhea in exam days. It was more, but it subsided a bit.” (Student 17, Female)

“It’s not psychological, but I have acid reflux and can’t eat from stress. I go home in the holidays and never have it for 1,5 months. Here, I get it 3-4 times each week.” (Student 12, Male)

“I have migraine. I never had it till I came here.” (Student 13, Male)

“Loss of appetite.” (Student 12, Male)

In addition to their psychological problems, international students encountered complications due to social security regarding admission to a health service and treatment.

“Since we take scholarships, we pay (Social Security Service - SSS) premiums.” (Student 5, Female)

“I have private insurance, but outpatient only.” (Student 7, Male)

“Not to be sent from one place to another, I get sick but don’t go (to the physician). I heal myself with natural products.” (Student 21, Male)

### Goal commitment/institutional attachment

Goal Commitment/Institutional Attachment is generally considered as a part of academic adjustment or social adjustment. In this section of the findings, examples that contain only goal commitment/institutional attachment are given.

International students did not report any positive experiences on the topic. One of the main problems appears to be the lack of communication between the students and the management. Students wanted a mechanism where they are notified and can gain information.

“… the school decides on something, and nothing changes whether we object to or not, we have difficulty in being notified. The number (of international students) is so low.” (Student 18, Male)

“We had a classroom teacher in high school. If we had a problem, we went to him. Here (at medical school) we have a counselor but we never saw him. We don’t know who to go to. I am a freshman and have friends at 6th year, and they say we didn’t see him yet, how couldn’t you?” (Student 13, Male)

The international students also compare universities in providing solutions to their problems. This affects the process of Goal Commitment/Institutional Attachment negatively.   With the effort of easing adjustment, they compare applications in different universities, but become unhappy in the end.

“I don’t know how true it is, but they don’t like strangers in our university. (A friend of mine) studies at the Faculty of Dentistry at the X University. (According to him) the international students easily go to the tutors. The tutors even ask if they have a problem when they meet.” (Student 12, Male)

“… I had a friend in X University. He had a counselor… he (the counselor) would always come by and ask. (the counselor) would call him to see if he had any trouble. When I came to Ege, I heard there was a counselor, but never met such a thing. No one asks (about me) and I don’t even know who he/she is.” (Student 1, Male)

Another area of problems negatively affecting Goal Commitment/Institutional Attachment is the issue of social security for international students. When the students encounter a health problem, despite studying at a medical school, they cannot find institutional solutions.

I got the flu and couldn’t stand. I went to student affairs and (since I was in medical school) told them I wanted to be examined as a member of the personnel. They told me to write a petition. Then they asked me for a student card. Despite being a student, since I wasn’t a part of the Turkish social security system, they sent me from one place to another. I couldn’t stand, so I gave up and returned home (Student 13, Male).

As a result, the students were found to use the coping strategies of acceptance, self-blame, use of instrumental and emotional support, active coping, positive reframing, behavioral disengagement, self-distraction, and substance use regardless of adjustment areas. For this reason, the use of qualitative data categories were (adjustment areas and coping strategies) interpreted together. In cases where they had a potential to cause change, the students were found to use functional coping strategies (such as changing place of accommodation, studying habits, and/or course materials, or choosing to study instead of attending a social activity and spending money tightly); and in cases where they didn’t have such potential, students were found to prefer goal-oriented reinterpretation strategies (nonfunctional coping strategies).

## Discussion

In this study, the adaptation and coping strategies of undergraduate international medical students to university life in Turkey were examined. The problems encountered by the students during the adaptation process were determined, and various suggestions were introduced to solve these problems.

Study findings have shown that international medical students have come to Ege University School of Medicine from seven different regions of the world. Turkey, being a transitory country between Europe and Asia, has become a conscious choice for international students for her geographical location to pursue their educational goals. In addition to this, the education programs of the universities in Turkey being compliant especially with that of European universities (due to the Bologna process) also appears as a determining factor in their choice of university.

Qualitative and quantitative data on academic adjustment have shown that international students aiming to be proficient doctors, came to Turkey with a focus on education, and determined to complete their education despite all difficulties. The fact that the students couldn’t reach anticipated academic success was because of their academic and cultural differences despite studying hard. This seems to be in compliance with the Habitus concept of Bourdieu. Their departure from their own habitus and encounter with a new one also supports the “Les Rites the passage” concept of Gennep.^33^ The students, who stated that they got used to the conditions they lived in with time, cross the threshold in adjustment between habitus, and merge with the new habitus.

To illustrate, students experience the stage of separation from their families and culture in their freshman year, the threshold in the second and third years, and the integration into the new culture stage in their fourth, fifth, and sixth years. The integration stage, which the students termed as “we got used to it”, is the stage defined as an adjustment by Gennep and Lysgaard.^9^^-^^33^ The fact that international students mentioned success-oriented scholarship within the context of academic success also supports the validity of Maslow’s Hierarchy of Needs Theory with regard to the adjustment process. According to the Biopolitics and Biopower theory, able power has a say on the right of an individual to live and commute in a society. In summary, international individuals who do not obey the rules of society are forced into complying with the norm/status quo. Institutions such as jails, hospitals, and dormitories are established to serve this normalization process. Being an international student places one outside the norms of society, but staying at a dormitory lets someone comply with the norms more easily. However, students who love their freedom ignore the issue of compliance with the norms and prefer to live in apartments rather than dormitories. The freedom experienced by a student staying at an apartment is, in a way, an effort to keep one’s own habitus. However, this effort causes difficulties in the academic adjustment area.

The quantitative and qualitative data regarding Academic Adjustment showed that students wanted to be good physicians, came to Turkey for this reason, and they want to complete the education against all hardships. Despite a lot of effort, they cannot achieve the academic success they expect due to hardships brought about by differences. Nevertheless, language barriers remain some of the most reported hindrances that physicians need to overcome in a healthcare setting.^1^^-^^4^^-^^7^^-^^34^^,^^35^ It was also discovered that motivations of the students were high, they made a great effort, and were more flexible in adjusting to the academic environment, but they did not reach the expected level of academic success. Students stated that in time they got used to the conditions they had trouble adjusting to in the beginning, and their success increased with passing years. The “getting used to in time” concept is consistent with the U Curve approach of Lysgaard.^9^

It was also found that Social Adjustment is the subscale of the Student Adaptation to College Questionnaire with the lowest mean score. International students are willing to participate in social activities that could positively affect social adjustment. The fact that the students prioritize academic success and not prefer to participate in social activities supports Maslow’s theory.

Moreover, prejudiced and discriminatory attitudes were found to have an effect on social adjustment. The Queer theory explains some of the problems experienced by international students.  Being the other, especially in their interactions with academic advisors, students point out that discriminatory attitudes come to the forefront. In studies by Dunne and McGarvey, the most difficult form of adjustment was stated to be a social adjustment. In Dunne’s study, it was stated that there were difficulties in social adjustment in a manner similar to our study.^3^^-^^36^ However, the point where our study differs from Dunne’s is the fact that international students have good relations with domestic students at Ege University.^36^ The theories of Habitus and Liminality explain how the students are/function in a different social structure, and how problems arise from language, culture, race, politics,  education system, and the longing they have for their families. The international students use different coping strategies to get accustomed to the new habitus and cross the threshold. They term these strategies as “we get used to it in time.” Historical myths and nationalistic discourses have also been found to affect the social adjustment of international students negatively. The adjustment problems based on historical myths appear to emerge from the attitudes of domestic students mostly. International students view this as discrimination. In contrast, participants in Sabbadini’s study identified their classmates as one of the most valuable sources of help and support.^7^

Hence, in this study, the physiological adjustment of the students was found to be better than their psychological adjustment. In Personal/Emotional Adjustment, the fear of not reaching education goals increases the visibility of its psychological effects on students. While students make an effort to solve their physiological problems, they use nonfunctional coping strategies such as acceptance when it comes to psychological issues. The problems encountered during Personal/Emotional adjustment can be explained via two theories. The psychological and physiological health problems can be thought to emerge from the habitus change of the students. The fact that the students had no local health insurance and couldn’t benefit from health services can be explained by being queer.

As to Goal Commitment/Institutional Attachment, it is not termed alone and is generally considered as a part of Academic Adjustment or Social Adjustment. Although a student comes to the institution to fulfill a specific academic goal, various situations regarding the institution can affect his/her overall adjustment process (the combination of Goal Commitment/Institutional Attachment and Academic Adjustment). Although international students choose to come to Ege University School of Medicine, there are no institutional mechanisms to ease Social Adjustment (the combination of Goal Commitment/Institutional Attachment and Social Adjustment) for them. Thus, the expectations of the students from education management cannot be met, and the students again use nonfunctional coping strategies.

According to the social model, exhibiting differences between students is performed not to point out the other but to determine needs. Therefore, it could be possible for individuals who have needs that differ from one another to live together in the same society. According to the social model, management power has a determining role in the formation of a social structure appropriate for the social model. Interventions such as education managements establishing a bureau specific to international students or meeting their health service needs on campus are critical in combating the students’ adjustment problems.

With the lack of such mechanisms, international students were observed to use the coping strategies of acceptance, self-blame, use of instrumental and emotional support, active coping, positive reframing, behavioral disengagement, self-distraction, and substance use all of which were mentioned in the Brief COPE – as supported by previous research findings.^4^^-^^12^^,^^13^^-^^18^ However, despite having a mean score of above 5 in Brief COPE, the coping strategies of planning, self-blame, self-distraction, and religion were not found in the focus group interviews. The reason behind this would be that the focus group interviews were informal and problems and solutions were expressed with examples. The focus group interview data is obtained from experience. However, the quantitative data obtained via the Brief COPE provides data on what international students would do in hypothetical situations. For example, even though the planning coping strategy took the highest mean score in quantitative data, it was not mentioned in focus group interviews. This shows that the student has an inclination to plan but cannot translate it into real life. Similarly, the denial and humor strategies were not termed in qualitative data, either.

### Limitations

International students’ not being able to use Turkish effectively has caused the transcript consist of many short and inverted sentences. Self-administered questionnaire technique was used in this study. Social desirability bias is yet another limitation in this study.

## Conclusions

In this study, the university adaptation of undergraduate international medical students were evaluated. It can be said that for the individual (international medical student) to be healthy and happy, and for the faculty to be productive, different cultures should co-exist in harmony. According to the findings of this study, in order to increase and ease the adaptation of international medical students to university life, and to minimize problems, the following suggestions were developed: first, before international medical students start their education, their needs potentially affecting their adaptation process should be determined. For this purpose, feedback mechanisms should be established and used effectively. Second, orientation training should be planned and implemented for international medical students. Third, language problems encountered by students should also be analyzed in a detailed manner. Especially when planning exams, culturally variable idioms (straw instead of yellow, etc.), terms, and synonyms should be considered concerning the academic success and reading speed of an international student. Fourth, an international student bureau should be established on the campus and should take an active role in solving bureaucratic problems international students might face. And finally, the University Counseling and Guidance Service and peer support systems should be used more effectively. International students should be encouraged to participate in activities designed to help them mingle with domestic students.

To conclude, this study has pointed out the critical issues in supporting international students in adapting to university life in Turkey. Globalization, the Bologna Process, and similar developments have increased the desire to receive high-quality education beyond borders and work under better conditions. The influence of globalization may remain insufficient in resolving the issues that arise when different cultures meet. The problems encountered by international students as a whole may be considered as an example of this. Revising the content of education programs to enable international exchange is also not sufficient enough by itself to meet the needs of international students. Future studies should be planned to resolve the cultural issues as well as the issues of adaptation to university life in Turkey.

### Acknowledgements

We thank Hanife Ankara, an employee at the Medical Education Department, whose help and valuable input made the project possible. We also thank our international students who participated in the study, and showed their support sincerely.

### Conflict of Interest

The authors declare that they have no conflicts of interest.
